# Physicochemical and Microstructural Analyses of Pepsin-Soluble Collagens Derived from Lizardfish (*Saurida tumbil* Bloch, 1795) Skin, Bone and Scales

**DOI:** 10.3390/gels8080471

**Published:** 2022-07-27

**Authors:** Abdul Aziz Jaziri, Rossita Shapawi, Ruzaidi Azli Mohd Mokhtar, Wan Norhana Md. Noordin, Nurul Huda

**Affiliations:** 1 Faculty of Food Science and Nutrition, Universiti Malaysia Sabah, Kota Kinabalu 88400, Malaysia; azizjaziri@ub.ac.id; 2 Faculty of Fisheries and Marine Science, Universitas Brawijaya, Malang 65145, Indonesia; 3 Borneo Marine Research Institute, Universiti Malaysia Sabah, Kota Kinabalu 88400, Malaysia; rossita@ums.edu.my; 4 Biotechnology Research Institute, Universiti Malaysia Sabah, Kota Kinabalu 88400, Malaysia; ruzaidi@ums.edu.my; 5 Fisheries Research Institute, Batu Maung 11960, Malaysia; norhana@dof.gov.my

**Keywords:** lizardfish by-product, pepsin-soluble collagen, structural characteristics, biochemical properties

## Abstract

Reducing food waste is critical for sustainability. In the case of fish processing, more than sixty percent of by-products are generated as waste. Lizardfish (*Saurida tumbil* Bloch, 1795) is an economically important species for surimi production. To address waste disposal and maximize income, an effective utilization of fish by-products is essential. This study aims to isolate and characterize pepsin-soluble collagens from the skin, bone and scales of lizardfish. Significant differences (*p* < 0.05) in the yields of collagen were noted with the highest yield recorded in pepsin-soluble skin collagen (PSSC) (3.50 ± 0.11%), followed by pepsin-soluble bone collagen (PSBC) (3.26 ± 0.10%) and pepsin-soluble scales collagen (PSCC) (0.60 ± 0.65%). Through SDS–polyacrylamide gel electrophoresis, the presence of two alpha chains were noted and classified as type I. From Fourier transform infrared spectroscopy (FTIR) analysis, the triple-helix structure of the collagen was maintained. The X-ray diffraction and UV visible spectra characteristics of the lizardfish collagens in this study are similar to the previously reported fish collagens. In terms of thermostability, PSSC (*T_max_* = 43.89 °C) had higher thermostability in comparison to PSBC (*T_max_* = 31.75 °C) and PSCC (*T_max_* = 30.54 °C). All pepsin-soluble collagens were highly soluble (>70%) in acidic conditions (particularly at pH 4.0) and at low sodium chloride concentrations (0–30 g/L). Microstructural analysis depicted that all extracted collagens were multi-layered, irregular, dense, sheet-like films linked by random coiled filaments. Overall, pepsin-soluble collagens from lizardfish skin, bone and scales could serve as potential alternative sources of collagens.

## 1. Introduction

To date, around 1.3 billion tons of food waste is discarded annually, representing 30 percent of total edible food [1]. Reducing food waste is the main concern of the United Nation Sustainable Development Goals (SDGs), specifically under Sustainable Consumption and Production (Goal 12). Global seafood consumption increased from 72 million tons in the 1980s to 156 million tons in 2018 [2]. In seafood processing, approximately 60 percent of the whole fish by weight is considered a by-product and may be discarded as waste [3]. On the other hand, by-products are rich in organic matter and perhaps should cost more to dispose of. Moreover, environmental pollution associated with seafood processing waste is causing increasing concern and monetary burdens for manufacturers [4]. An effort to transform seafood by-products into high-value-added goods, such as collagen and its derivative (gelatin), is strongly required. Collagen makes up around 30 percent of the protein in mammals and is a prominent structural component of the extracellular matrix. It has a typical right-handed triple helix with three polypeptide chains. Each chain is mostly constructed of glycine–proline–hydroxyproline repeats [5]. To date, approximately 29 types of collagen, named types I–XXIX, have been documented based on the protein structure, amino acid sequence and function [6]. As a valuable commodity, collagen, particularly type I, is widely used in food, cosmetics, pharmaceuticals and biomedicine. In the food industry, it serves as a colloidal stabilizer, emulsifier and foaming agent [7]. In cosmetics, it is broadly used as a natural humectant and moisturizer for the skin to prevent aging [8]. Additionally, collagen plays important roles in accelerating wound healing, developing drug delivery systems, tissue engineering and treating hypertension, obesity and diabetes [9]. Commercial collagens (type I) are generally produced from land-based animals, i.e., cattle, pigs and poultry [10]. However, there are health concerns related to land-based animal collagen, especially its association with the possible transmission of diseases, such as foot and mouth disease, bovine spongiform encephalopathy and avian influenza. There is also a concern, particularly expressed by several religions. Hindus and Sikhs are not allowed to consume bovine-based collagen, and porcine-based collagen is absolutely forbidden among Jews and Muslims [11]. Thus, efforts to explore alternative sources of collagen are urgently needed.

Over the last decade, fish collagen has received considerable attention as a potential alternative form of collagen due to being safer and acceptable to most religions. Additionally, its characteristics are comparable to collagen isolated from land-based animals [12]. The characteristics of collagens extracted from fish by-products (skin, bone and scales) have been well studied, For example, bigeye tuna (*Thunnus obesus*) scales, bone and skin collagen [13]; grass carp (*Ctenopharyngodon idellus*) bone, scales and skin collagen [14]; the bone and skin collagen of Spanish mackerel (*Scomberomorous niphonius*) [15]; sturgeon (*Huso huso*) skin collagen [16]; barramundi (*Lates calcarifer*) skin collagen [17]; tiger grouper (*Epinephelus fuscguttatus*) skin collagen [18] and carp (*Cyprinus carpio*) scale collagen [19]. To date, extractions of fish collagen have been aided by acids and proteolytic enzymes. However, the acid-aided technique yielded less collagen, required a long period of time to perform and generated substantial undissolved collagen residues [13]. Another technique is the enzyme-aided method and pepsin was predominantly used because of its ability to cleave cross-linked molecules and peptides in the telopeptide region without changing the triple helical structure of the targeted collagen [20]. Generally, this process is performed under acidic conditions to increase the extractability efficiency of the sample, resulting in an increase in solubilized collagen and reduces unextracted materials [15].

In Malaysia, one of the most important fish used as raw materials for surimi and surimi-based products is lizardfish (*Saurida tumbil* Bloch, 1795). This is due to its high gel strength, high production yield, white flesh, affordability and common availability [21,22]. Morphologically, lizardfish has a subcylindrical and elongated body with a total length between 19 and 35 cm; its body color is light brown dorsally, silvery white ventrally and black elsewhere with faint crossing bands [23]. In Malaysia, about 48,153 metric tons of lizardfish were caught from 2015 to 2019 [24]. As previously mentioned, a large quantity of by-products was generated during fish processing, including skin, scales, bones, fins, heads and viscera. The utilization of by-products from lizardfish, particularly the skin, bones and scales, could be utilized to the maximum. The production of acid-soluble collagens from the skin, bone and scales of lizardfish and their physicochemical properties have been evaluated in our previous works [25,26,27]. This study was conducted to further extract collagen from the undissolved matter from acid-aided collagen-extraction studies by using the pepsin enzyme in order to maximize the output. The microstructural and biochemical characteristics are also determined.

## 2. Results and Discussion

### 2.1. Yield and Hydroxyproline Composition

Three types of undissolved matter from the lizardfish skin, bone and scale acid-aided collagen-extraction process were further extracted with pepsin and the collagen yields are tabulated in Table 1. The pepsin-soluble collagen from the scales (PSCC) were significantly (*p* < 0.05) the lowest (0.6%) compared to the skin (PSSC) and bones (PSBC). Other studies also reported a low yield of pepsin-soluble collagen from the scales of fish, including tilapia (*Oreochromis niloticus*) (0.71%) [28], seabass (*L. calcarifer*) (1.06%) [29], bighead carp (*Hypophthalmichthys nobilis*) (1.1%) [30] and spotted golden goatfish (*Parupeneus heptacanthus*) (1.2%) [31]. The reason might be due to the composition of fish scale, which is highly ordered type-I collagen fibers with many cross-linked areas and hydroxyapatite components (Ca_5_(PO_4_)_3_OH) [32]. However, the PSCC (0.60 ± 0.06%) in the present study was 3-fold greater than the acid-soluble collagens (ASCs) (0.18 ± 0.03%) from our previous result in [27]. The pepsin enzyme could have efficiently cleaved the cross-linked area, particularly at the telopeptide area of collagen [33]. Additionally, a higher collagen yield was marked in the PSBC sample (3.26 ± 0.10%) compared to the acid-extracted collagen (1.73 ± 0.08%) from the bone of lizardfish [25]. This finding was in accordance with the study of fish-bone collagen from grass carp (*C. idellus*) [14] and Spanish mackerel (*S. niphonius*) [15]. However, for lizardfish skin, the pepsin-aided treatment yielded a lower collagen percentage (3.50 ± 0.11%) than the acid-aided process (11.73 ± 1.14%), as reported in our previous work [26], and other fish, including sturgeon (*H. huso*) [16], sailfish (*Istiophorus platypterus*) [34] and Spanish mackerel (*S. niphonius*) [15]. It was suggested the acid solutions increased the extractability of solubilized skin collagen, resulting in less collagen in the undissolved matter, and the final PSC obtained was also lower. The overall total yields of lizardfish collagen isolated from the skin, bone and scales were 15.23%, 4.99% and 0.78%, respectively. Thus, the use of the pepsin enzyme could further increase the chance of maximizing the quantity of collagen extracted from lizardfish by-products.

Hydroxyproline (Hyp) plays an essential role in stabilizing the triple helical structure of collagen and a prominent component of imino acid attributed to collagen molecule. Therefore, the Hyp content reflected the real amount of collagen in living organisms since it is present exclusively in collagen [28]. In this study, the Hyp content in PSSC, PSBC and PSCC was 85.71 ± 0.13 mg/g, 84.85 ± 1.38 mg/g and 81.72 ± 0.47 mg/g, respectively. The highest value was recorded in the PSSC sample. The Hyp content for PSSC and PSBC was significantly greater (*p* < 0.05) than PSCC. These results are comparable to other pepsin-soluble fish by-product collagens, including golden pompano (*Trachinotus blochii*) skin (Hyp = 73.7 mg/g) and bone (Hyp = 77.5 mg/g) [7], Spanish mackerel (*S. niphonius*) skin (Hyp = 68.7 mg/g) [15], Nile tilapia (*O. niloticus*) skin (86 mg/g) [35], sturgeon fish (*H. huso*) skin (Hyp = 80.3 mg/g) [16], bigeye tuna (*T. obesus*) skin (Hyp = 82.38 mg/g) [13], grass carp (*C. idellus*) bone (Hyp = 77 mg/g) and scales (Hyp = 76 mg/g) [14], black drum (*Pogonia cromis*) bone (Hyp = 84.6 mg/g) and scales (Hyp = 87.9 mg/g) [36], bighead carp (*H. nobilis*) bone (Hyp = 73.5 mg/g) and scales (Hyp = 56.2 mg/g) [30] and seabass (*L. calcarifer*) (Hyp = 89 mg/g) [29]. Furthermore, the total collagen composition derived from the lyophilized collagen products was determined by multiplying the amount of Hyp with a conversion factor of 7.7, as stated by Kittiphattanabawon et al. [37]. The results show that the total collagen (mg/g) obtained corresponds with the amount of Hyp in the samples. Fish species, age, size, composition, structure of fish tissue and extraction process were demonstrated to influence the Hyp content and yields [38].

### 2.2. Color Attributes

The color of pepsin-extracted collagens of the skin, bone and scales of lizardfish was determined (Table 1). The L* value of all pepsin-soluble collagens was significantly different (*p* < 0.05), with a greater value marked in PSCC, followed by PSBC and PSSC. For redness (a*) and yellowness (b*) attributes, PSSC and PSCC, respectively, had the highest value. Collagen produced from the bone (PSBC) exhibited the highest whiteness index (WI), although not significantly different (*p* > 0.05) to the scales collagen (PSCC). In comparison to seabass (*Lates calcarifer*) skin collagen (L* = 65.41 and WI = 65.27) [39], PSBC and PSCC were lighter and whiter. Additionally, the L* and WI of extracted collagens in the present study were lower than the H_2_O_2_-treated snakehead (*Channa argus*) skins (L* = 89.49 and WI = 88.09). The use of hydrogen peroxide might have removed the sample’s pigment during the pre-treatment process [40]. In general, lighter-colored collagen is preferred so that it does not override the original color of the finished products [41].

### 2.3. SDS-PAGE Analysis

**Figure 1:**
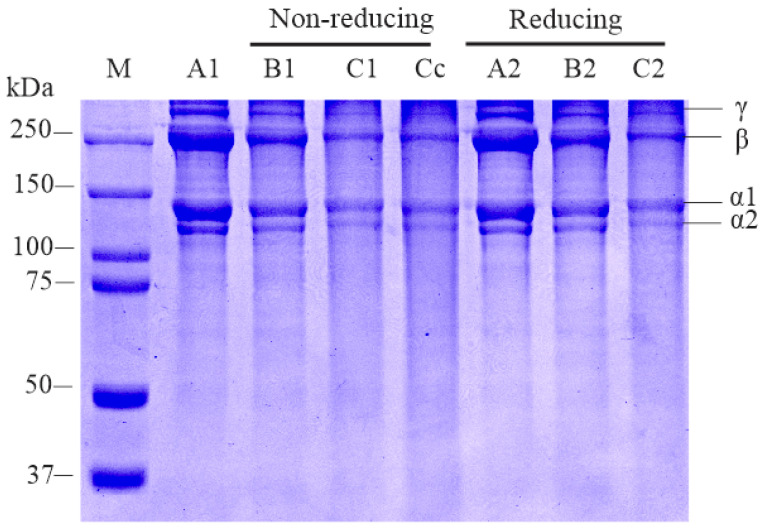
Electrophoretic characteristic of pepsin-solubilized collagens from lizardfish skin, bone and scales confirms the specificity of band patterns consisting of α1 (128.8 kDa), α2 (113.5 kDa), β (250 kDa) and γ (345.2 kDa) isomers. M: protein marker; Cc: commercial calf-skin collagen (standard); A1 and A2: pepsin-soluble skin collagen (PSSC); B1 and B2: pepsin-soluble bone collagen (PSBC); C1 and C2: pepsin-soluble scales collagen (PSCC).

All pepsin-soluble collagens, i.e., PSSC, PSBC and PSCC, presented similar electrophoretic band patterns, containing two alpha (α1 and α2), β- and γ-chains. The molecular weights (MWs) of each chain were estimated at 128.8 kDa and 113.5 kDa, respectively (Figure 1). The pepsin-extracted lizardfish collagens in the present study were type-I collagen based on the findings of Benjakul et al. [42] who classified collagen with two α chains as type-I collagen. These results are in accordance with the collagen extracted from loach (*Misgurnus anguillicaudatus*) (α1 = 127 kDa and α2 = 115 kDa) [43], Nile tilapia (*O. niloticus*) (α1 = 125 kDa and α2 = 114 kDa) [35], seabass (*L. calcarifer*) (α1 = 118 kDa and α2 = 105 kDa) [29] and golden pompano (*T. blochii*) (α1 = 120 kDa and α2 = 100 kDa) [7]. Meanwhile, other chains (β = 250 kDa and γ = 345.2 kDa) in all extracted collagens represented dimer and trimer bands, which were also detected in other fish collagens [28,44]. In terms of band intensity, the PSSC sample showed the greatest intensity compared to PSBC, PSCC and even commercial calf-skin collagen (standard). It can be argued that the pepsin-extracted collagen from the skin of lizardfish had a higher proportion of intra- and inter-crosslinks [33]. On the other hand, no differences in electrophoretic patterns were detected in PSSC, PSBC and PSCC under reducing (with β-ME) and non-reducing (without β-ME) treatments, indicating no disulphide bonds (R−S−S−R′) in all extracted samples, as reported in the literature [28,29].

### 2.4. UV-Vis Absorption

**Figure 2:**
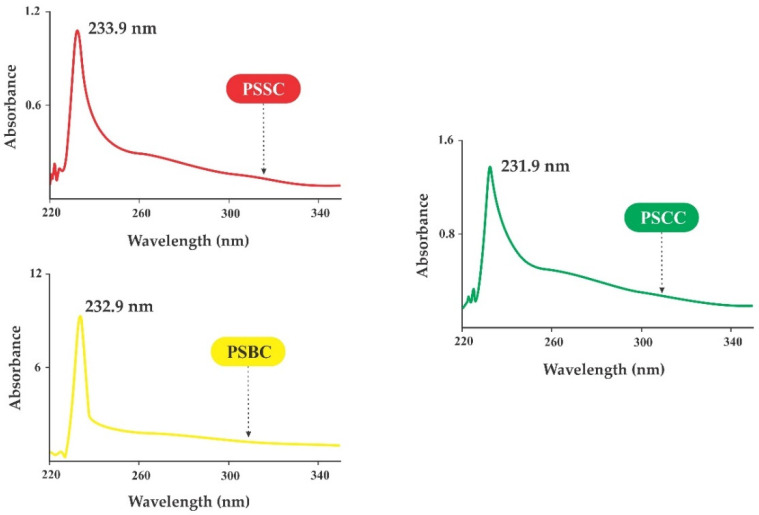
UV-visible absorption spectra of pepsin-soluble collagens from the skin, bone and scales of lizardfish. PSSC: pepsin-soluble skin collagen; PSBC: pepsin-soluble bone collagen; PSCC: pepsin-soluble scales collagen.

Figure 2 shows the spectra of three pepsin-solubilized collagens from lizardfish by-products. Generally, the maximum absorption spectrum detected was located at 210–240 nm [45]. In the present study, the maximum spectra were recorded at 233.9 nm, 232.9 nm and 231.9 nm for PSSC, PSBC and PSCC, respectively. The results confirm that the absorption peaks found in all type-I collagens from lizardfish by-products are associated with the functional groups of carboxyl (-COOH), amides (CONH_2_) and carbonyl (C=O), which belong to the polypeptide chains of fish collagen [46]. On the other hand, the low absorption peaks detected in all pepsin-soluble collagens were presented at wavelengths between 300 nm to 250 nm, representing aromatic amino acids, such as phenylalanine (Phe), tryptophan (Trp) and tyrosine (Tyr). Moreover, our previous work on lizardfish collagen extracted with acids presented the prominent peak at 230–231 nm and was in agreement with other species, such as northern pike (*Esox lucius*) [47], miiuy croaker (*Miichthys miiuy*) [45], red drum (*Sciaenops ocellatus*) [48] and puffer fish (*Lagocephalus inermis*) [49].

### 2.5. Attenuated Total Reflection–Fourier Transform Infrared Spectroscopy (ATR–FTIR)

**Figure 3:**
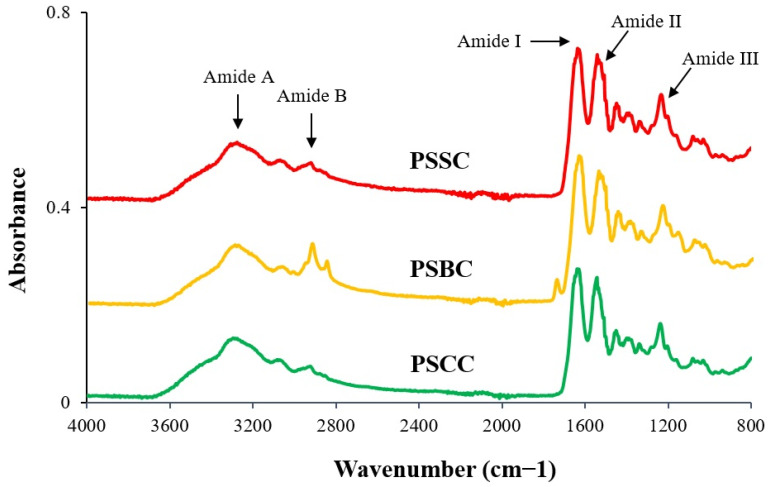
IR spectra of pepsin-soluble collagens from the skin, bone and scales of lizardfish. PSSC: pepsin-soluble skin collagen; PSBC: pepsin-soluble bone collagen; PSCC: pepsin-soluble scales collagen.

The analysis of PSSC, PSBC and PSCC using FTIR showed various absorption peaks consisting of amides I–III, and amides A and B, as depicted in Figure 3 and described in Table 2. At the location of amides I–III in particular, it could be used for the clarification of the collagen triple helix, as previously described by many researchers. The Δ*v(v_I_-v_II_)* formula, where the difference in wavenumber (cm^−1^) between amides I and II was less than 100 cm^−1^, indicated that the triple helical structure of the extracted collagen in a recent study was preserved [50]. As a result, the Δ*v* value of the lizardfish collagen (i.e., PSSC, PSBC and PSCC) was below 100 cm^−1^, representing 95.05 cm^−1^. Thus, it could be assumed that the structure of the collagen triple helix derived from the skin, bone and scales of lizardfish was maintained. Another approach in verifying the triple helical structure of fish collagen is by using the ratio of amide III and the 1450 cm^−1^ band (AIII/A1450), as proposed by Doyle et al. [51]. The data revealed that the triple helical structures of PSSC, PSBC and PSCC were intact during the extraction process, as presented by their absorption ratio values (~1.0). Overall, all the absorption peaks (amides I–III, and amides A and B) observed in this study are in agreement with the collagen from bigeye tuna (*T. obesus*) skin, bone and scales [13]; loach fish (*M. anguillicaudatus*) skin [43]; golden pompano (*T. blochii*) skin [47] and tilapia (*O. niloticus*) scales [28].

### 2.6. Evaluation of X-ray Diffraction (XRD)

**Figure 4:**
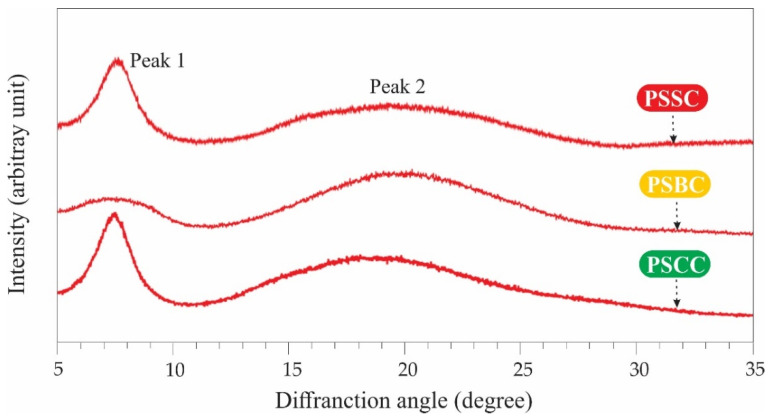
XRD of pepsin-soluble collagens from lizardfish skin, bone and scales. PSSC: pepsin-soluble skin collagen; PSBC: pepsin-soluble bone collagen; PSCC: pepsin-soluble scales collagen.

The X-ray diffraction graph of the PSSC, PSBC and PSCC samples is illustrated in Figure 4. Two significant peaks can be observed, i.e., sharp (peak 1) and broad (peak 2) peaks located at the diffraction angles (2θ) of 7.00–7.65° and 19.24–21.24°, respectively. Although the PSBC sample showed a slightly different peak 1 pattern, it still generally represents a collagen triple-helix structure as observed in commercial calf-skin collagen [27]. The differences in the peaks might be due to sample preparation that was arranged in a random order. Moreover, our previous reports on acid-extracted collagens also exhibited two diffraction peaks [25,26,27] and other previous reports of carp (*C. carpio*) scale collagen [52], golden pompano (*T. blochii*) skin and bone collagen [7] and tilapia (*O. niloticus*) skin collagen [53]. Furthermore, d(Å) was used to predict the minimum value of repeated spacing d (Å). For the first peak, the distance between the molecular chains of the collagen triple helical structure in all pepsin-soluble collagens was slightly lower (d = 11.54–12.62 Å) than standard collagen (d = 12.00 Å) [27]. The d values of the extracted collagens’ second peak were between 4.18 Å and 4.61 Å, also relatively lower compared to that of reference (d = 4.58 Å), and this peak represents the distance between skeletons. The diameter (d) of a collagen molecule with a single left-handed helix chain and a triple helix structure is consistent with the diameter of isolated collagen from lizardfish skin, bone and scales. Overall, the lizardfish collagens extracted with pepsin had an undenatured triple helical structure and were in their native conformations.

### 2.7. Thermostability Analysis

**Figure 5:**
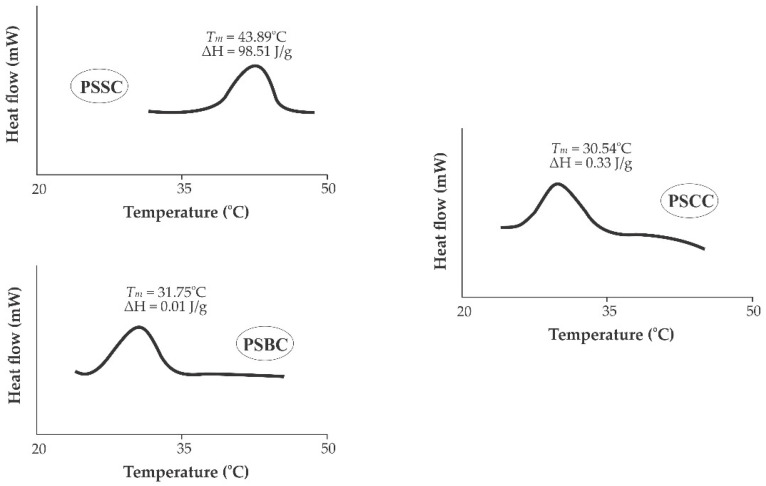
DSC thermogram of pepsin-soluble collagens from lizardfish skin, bone and scales. PSSC: pepsin-soluble skin collagen; PSBC: pepsin-soluble bone collagen; PSCC: pepsin-soluble scales collagen.

Using differential scanning colorimetry (DSC), the *T_max_* and Δ*H* values of pepsin-solubilized collagens were evaluated. The PSSC sample had the highest maximum transition temperature (*T_max_* = 43.89 °C) in comparison to PSBC (*T_max_* = 31.75 °C) and PSCC (*T_max_* = 30.54 °C) (Figure 5). The collagen with a higher *T_max_* value implied a higher thermostability with a higher Hyp content, as indicated in Table 1. The thermostability of the collagen triple helix was brought about by the imino acids (proline and hydroxyproline), particularly at pyrrolidine rings that were partially governed by hydrogen bonding via the hydroxyl group of Hyp [42]. In addition, Hyp served to stabilize the triple helical structure through the hydrogen bonding in coil-coiled alpha chains [54]. The variations in the thermostability of pepsin-soluble fish collagens were widely reported in seabass (*L. calcarifer*) skin (*T_max_* = 39.32 °C) [29], Spanish mackerel (*S. niphonius*) skin (*T_max_* = 14.66 °C) and bone (*T_max_* = 16.85 °C) [15], loach (*M. anguillicaudatus*) skin (*T_max_* = 33.61 °C) [43], tilapia (*O. niloticus*) scales (*T_max_* = 34.70 °C) [28], bigeye tuna (*T. obesus*) scales (*T_max_* = 31.63 °C) [13] and leather jacket (*O. niger*) bone (*T_max_* = 31–32 °C) [55]. For the Δ*H* point of view, the narrow areas under the thermogram peaks found in the PSSC, PSBC and PSCC samples reflect the low energy required to uncouple the alpha chains of lizardfish collagens and convert them into random coils. However, the difference in the *T_max_* and Δ*H* values of fish collagen depends on the composition of imino acid, extraction procedure and other environmental factors, such as water temperature, feed and habitat [37].

### 2.8. Microstructure Study

**Figure 6:**
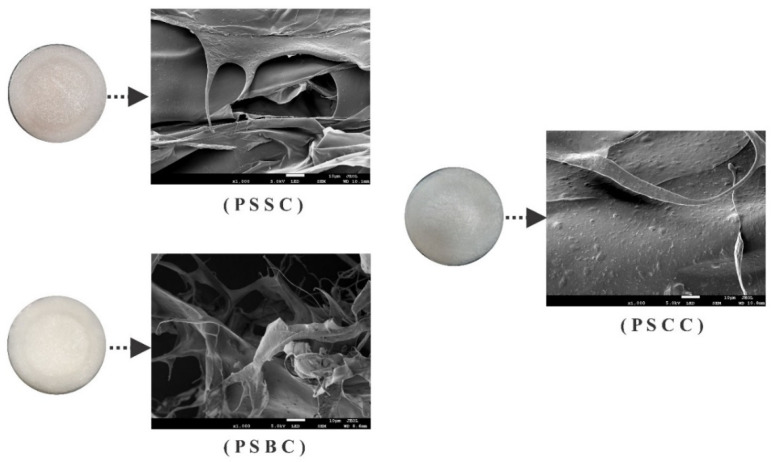
FESEM photograph of pepsin-soluble collagens from lizardfish skin, bone and scales. PSSC: pepsin-soluble skin collagen; PSBC: pepsin-soluble bone collagen; PSCC: pepsin-soluble scales collagen.

Figure 6 shows the microstructure of lyophilized pepsin-soluble collagens scanned by FESEM. The images of PSSC, PSBC and PSCC samples were mostly multi-layered, irregular, dense sheet-like films linked by random coiled filaments. Selected fibrillar and tubular structures were presented in all collagen samples. In addition, loose, porous and wrinkled structures were clearly visible at a magnification of 1000× because of dehydration during the lyophilization process, as described by Schuetz et al. [56]. Other fish collagens, including marine eel (*Evenchelys macrura*) skin [57], black ruff (*Centrolophus niger*) [58] skin and miiuy croaker (*M. miiuy*) [44] scales, have similar morphological structures to those presented in our present study. Previous findings have recommended collagens with fibrillary, interconnectivity and sheet-like film structures as a potential source of biomaterials in producing new biomedical and pharmaceutical products, including new wound dressing, skin and bone tissue formation, cell seeding material, cell growth, cell migration and coating application [9].

### 2.9. Solubility

**Figure 7:**
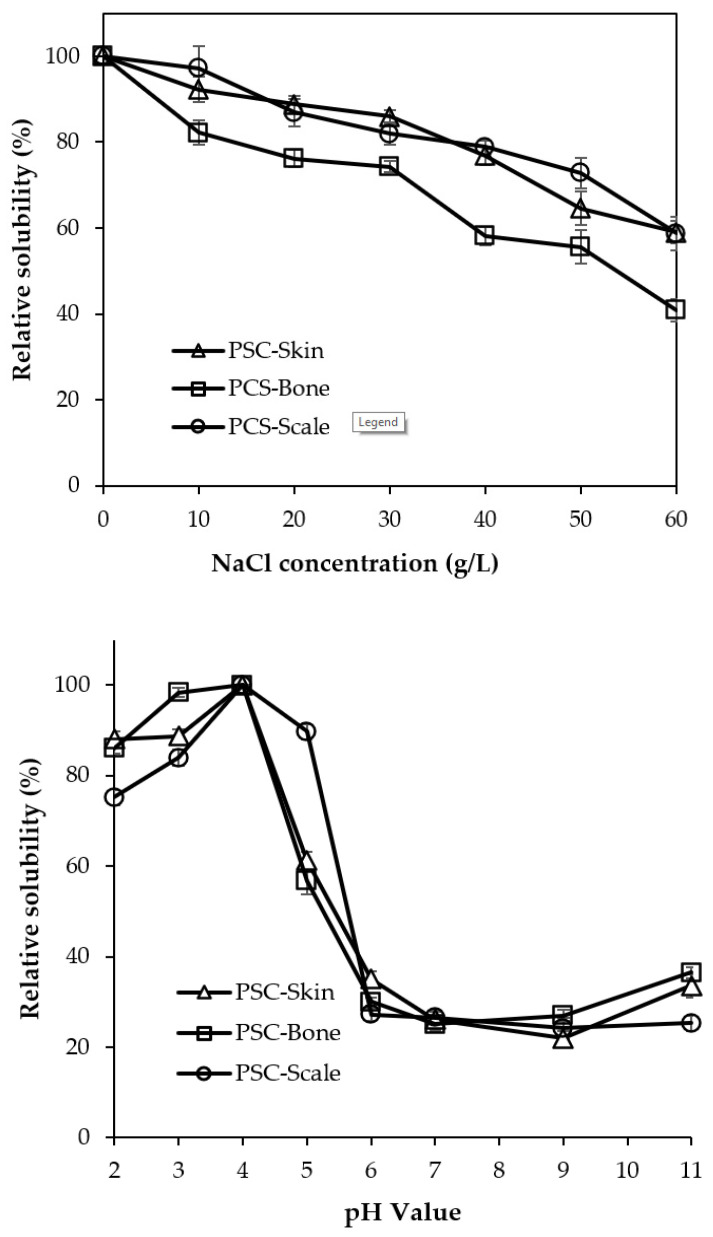
Relative solubility of pepsin-soluble collagens from lizardfish skin, bone and scales treated at different pH and NaCl concentrations. PSSC: pepsin-soluble skin collagen; PSBC: pepsin-soluble bone collagen; PSCC: pepsin-soluble scales collagen.

The solubility of PSSC, PSBC and PSCC samples at different pH and sodium chloride concentrations is depicted in Figure 7. All extracted collagens presented in this study were highly soluble in acidic conditions, with the highest solubility at pH 4.0. At a pH close to neutral (pH 6.0), the relative solubility (RS) in all samples decreased sharply (<35%). The lowest solubility was observed in alkaline conditions, particularly at pH 9.0. The obtained results could be explained by the hydrophobic–hydrophobic interactions among the collagen molecules that led to the total net charge becoming zero, particularly at the isoelectric point, which commonly occurs in slightly acidic and neutral conditions [20]. Our previous works on acid-extracted lizardfish collagen are in agreement with the present study, as well as pepsin-soluble collagen obtained from the skin and bone of Spanish mackerel (*S. niphonius*) [15], scales of barramundi (*L. calcarifer*) [29] and skin and bone of golden pompano (*T. blochii*) [7]. In the context of solubility in NaCl, all extracted collagens were tested at different concentrations (0–60 g/L). The high solubility of PSSC, PSBC and PSCC was clearly marked at a low NaCl level of 10–30 g/L. At more than 30 g/L, the solubility of each collagen sample gradually decreased. The results might have been influenced by the precipitation of the solubilized collagen. The increase in salt concentration hydrophobic–hydrophobic interactions within the polypeptide chains of collagen and competition for water subsequently generated protein precipitation [48], as demonstrated in several studies on pepsin-soluble collagens obtained from *P. heptacanthus* scales [31]; *H. nobilis* skin, bone, scales, fins and swim bladder [30]; and *T. blochii* skin [7].

## 3. Conclusions

Type-I collagen from the skin, bone and scales of lizardfish was produced from pepsin-assisted extraction. A higher percentage (based on wet-weight basis) of collagen was obtained from the skin (PSSC) rather than bone (PSBC), although not significantly (*p* > 0.05). More importantly, PSSC recorded the highest *T_max_* value in comparison to others, indicating that PSSC was more stable at high thermal conditions, probably due to the Hyp content. Moreover, the structures of the collagen triple helix of the skin, bone and scales of lizardfish were still preserved, as confirmed by the FTIR and XRD tests. In conclusion, the PSSC sample has more potential to be explored further as an alternative source of collagen due to its high yield and thermostability.

## 4. Materials and Method

### 4.1. Preparation of Lizardfish Skin, Bone and Scales

The lizardfish (*Saurida tumbil* Bloch, 1795) used in this study were obtained from a local fish market (Kota Kinabalu, Sabah, Malaysia) and then transported to the laboratory. To maintain their freshness, the fish were placed in a Styrofoam box with a 1:2 (*w*/*w*) ratio of fish to ice. Then, the fish were taxonomically identified and prepared for the separation of skin, bone and scales using a meat–bone separator (SFD-8, Taiwan). The length and weight of the fish samples were 29.86 ± 0.41 cm and 202.58 ± 16.05 g, respectively. The skin, bone and scales were then washed with running tap water. The skin and bone were cut to 1.0 × 1.0 cm^2^ and 0.5 × 0.5 cm^2^, respectively, with scissors (Brisscoes, Selangor, Malaysia). All processed samples were then placed into a polyethylene container and stored in a freezer (−20 °C) (Sharp, Tokyo, Japan) until experimentation.

### 4.2. Extraction of Pepsin-Soluble Collagen

**Figure 8:**
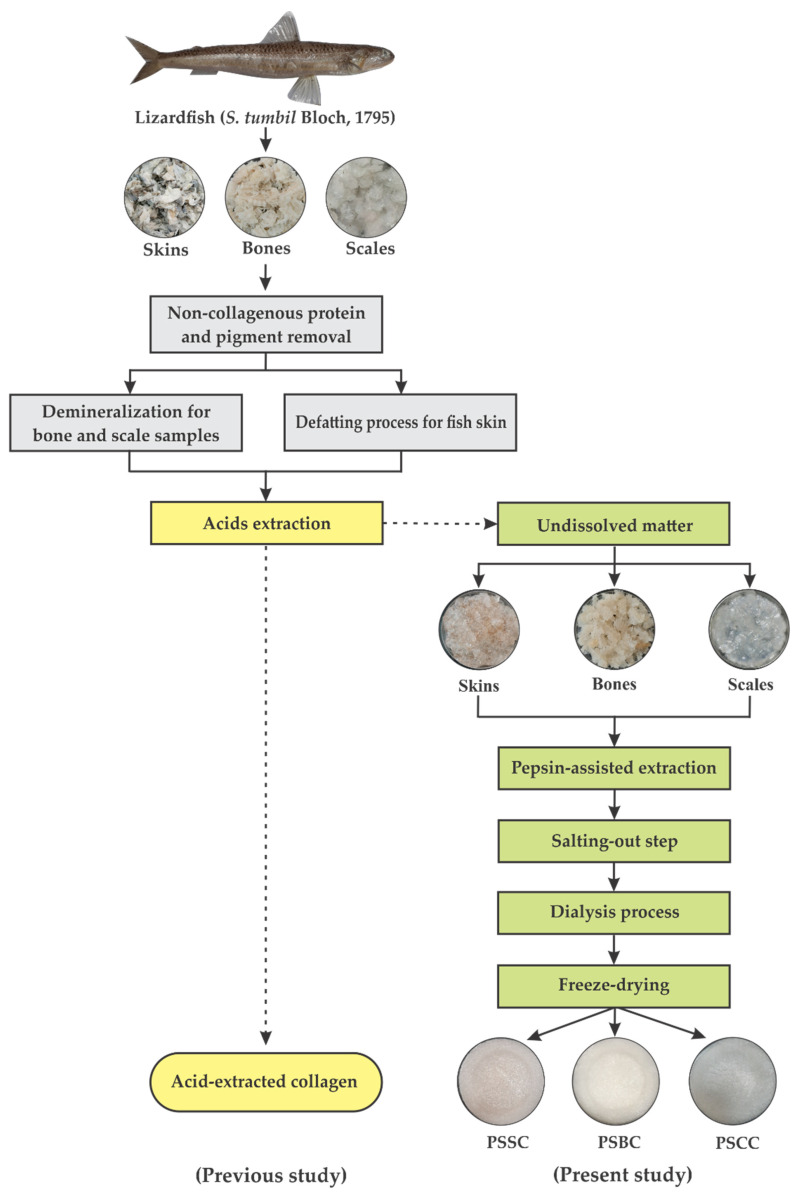
Extraction step of pepsin-soluble collagen from the skin, bone and scales of lizardfish. PSSC: pepsin-soluble skin collagen; PSBC: pepsin-soluble bone collagen; PSCC: pepsin-soluble scales collagen.

Pepsin-soluble collagen from the skin, bone and scales of lizardfish were prepared using the procedure developed by Jongjareonrak et al. [20] and Matmaroh et al. [31] with some modifications. The extraction processes were conducted in a chiller (4 °C) and the entire process of collagen production is illustrated in Figure 8. The prepared samples were soaked in 0.1 M NaOH at the ratio of 1:10 (*w*/*v*) for 6 h with continuous stirring to remove pigment and non-collagenous protein, and the NaOH solution was replaced every 3 h. The treated samples were washed with cold distilled water until reaching the neutral stage at pH 7.0. After alkaline treatment, fish bone and scales were subjected to demineralization by suspension in 0.5 M EDTA-2Na at a ratio of 1:10 (*w*/*v*) for 48 h, and the solution was changed every 16 h. The suspended samples were then washed with cold distilled water for 30 min, and the distilled water was replaced every 10 min. In terms of lizardfish skin, a defatting process was performed by adding 10% butyl alcohol at the ratio of 1:10 (*w*/*v*) for 24 h, and the solution was periodically changed every 12 h. In the present study, we used undissolved matter from our previous acids extraction processes [25,26,27]. All undissolved samples were isolated in an acidic condition by adding 0.5 M of acetic acid and 1.5% (*w*/*w*) of pepsin from a bovine origin (Himedia, Maharashta, India) at a solid–liquid ratio of 1:15 (*w*/*v*) for two days with continuous stirring. The solubilized samples were further separated using two layers of cheesecloth filtration. Then, the obtained supernatants were salted out by adding 2.5 M of sodium chloride (NaCl) containing a 0.05 M Tris base (NH_2_C(CH_2_OH)_3_) (Sigma-Aldrich, St. Louis, MO, USA) at a neutral condition (pH 7.0). Subsequently, the salted-out samples were centrifuged using a centrifuge machine (Eppendorf Centrifuge 5810R, Hamburg, Germany) at 15000× *g* for 20 min. The obtained pellets were subsequently dissolved with an acetic acid solution at the ratio of 1:5 (*w*/*v*). The solutions were then dialyzed through a tubing cellulose membrane avg. flat width 43 mm (1.7 in.) (Sigma-Aldrich, St. Louis, MO, USA) in 20 volumes of acetic acid solution (0.1 M) for 24 h, and changed with cool, distilled water for 48 h. The dialysates were then freeze-dried using a lyophilization machine (Labconco, Kansas City, MO, USA). After drying, the freeze-dried collagen samples named pepsin-soluble skin collagen (PSSC), pepsin-soluble bone collagen (PSBC) and pepsin-soluble scales collagen (PSSC) were kept at −20 °C until further analysis.

### 4.3. Analyses of Pepsin-Soluble Collagen

#### 4.3.1. Measurement of Yield and Hyp Content

Yields of PSSC, PSBC and PSCC were measured based on the wet weight of undissolved matter obtained from our previous works [25,26,27], and the formula is described below:

(1) $$\mathrm{Yield}\;\left(\%\right)=\frac{\mathrm{Weight}\;\mathrm{of}\;\text{freeze-dried}\;\mathrm{collagen}}{\mathrm{Weight}\;\mathrm{of}\;\mathrm{initial}\;\mathrm{wet}\;\mathrm{undissolved}\;\mathrm{matter}}\times \;100$$

The Hyp content of the extracted collagen was measured, as recommended by Bergman and Loxley [59]. The dried samples (PSSC, PSBC and PSCC) were subjected to hydrolysis in strong acid (6 M hydrochloric acid) at 110 °C for 24 h. Hydrolysates were then filtered using Whatman filter paper No. 4 (Sigma-Aldrich, St. Louis, MO, USA). Then, the filtrates were pH-adjusted with a 2.5 M NaOH until the pH was around pH 6.5. A total of 0.2 mL of the pH-adjusted samples was then transferred into prepared 15 mL tubes and 0.4 mL of isopropyl alcohol was added (Merck, Darmstadt, Germany). Subsequently, the solutions were mixed with 0.2 mL of prepared oxidant solution and incubated at room temperature for 5 min. After incubation, a total of 2.3 mL of Ehrlich’s reagent solution (previously prepared) was directly transferred and thoroughly mixed. The tubes were then placed in a water bath (Memmert, Schwabach, Germany) with a temperature of 60 °C for 25 min. The mixtures were subsequently cooled for 3–5 min in cooled tap water. Then, the mixtures were diluted with up to 10 mL of isopropyl alcohol. Absorbance against distilled water (dH_2_O) was determined at a wavelength of 558 nm using a spectrophotometer (Agilent Cary 60, Santa Clara, CA, USA). The Hyp standard solution (10 to 70 ppm) (Sigma-Aldrich, St. Louis, MO, USA) was also recorded.

#### 4.3.2. Color Measurement

The color parameter of pepsin-solubilized collagen from lizardfish skin, bone and scales was measured based on the procedure from a previous study [60]. We used a ColorFlex CX2379 (HunterLab, Reston, VA, USA) to measure the color attributes that are L* (lightness/brightness), a* (redness/greenness) and b* (yellowness/blueness). In addition, the whiteness index (WI) was also measured using the following equation from Briones and Aguilera [61]:

(2) $${\mathrm{WI}=100\;-\;[(100\;-\;L}^{*}{)}^{2\;}+\left(a^{*2}\right){+(b}^{*2}{)]}^{0.5}$$

#### 4.3.3. Sodium Dodecyl Sulfate-Polyacrylamide Gel Electrophoresis (SDS-PAGE)

The electrophoretic pattern of PSSC, PSBC and PSCC samples was measured using a Mini-PROTEAN electrophoresis system (Bio-Rad Laboratories, Hercules, CA, USA), as described by Laemmli [62]. About 2.5 mg of collagen samples were prepared by dissolving with a SDS solution and thoroughly mixed. The mixture was subsequently treated at a high temperature (85 °C) for 1 h. Then, the mixture was centrifuged at 8500× *g* for 5 min using a centrifuge (Eppendorf Centrifuge 5810R, Hamburg, Germany) to remove insolubilized matter. Following centrifugation, 15 µL of supernatants was transferred into a mini centrifuge tube, and subsequently 15 µL of sample buffer was added in the presence and absence of 10% β-mercaptoethanol (β-ME). The mixture was then subjected to heat treatment at the same temperature for 3 min and then loaded onto polyacrylamide gel, which contained both 4% stacking gel and 7.5% running (resolving) gel. During electrophoresis, a constant voltage of 120 volts for about 90 min was set to obtain a greater separation of protein bands. The acrylamide gel was then fixed for approximately 15 min using a prepared fixation solution. Then, the fixed gel was stained for around 10 min with the staining solution. The stained gel was further destained with 30% (*v*/*v*) methanol and 10% (*v*/*v*) acetic acid solutions. The electrophoretic bands were recorded and compared to the 10–250 kDa of the protein marker purchased from Bio-Rad Laboratories, Hercules, CA, USA.

#### 4.3.4. UV-Vis Spectra

Ultraviolet-visible absorption spectra of the PSSC, PSBC and PSCC samples were measured according to Zeng et al. [63]. A UV-Vis spectrophotometer (Agilent Cary 60, Santa Clara, CA, USA) was used in this study. Around 10 mg of the collagen sample was dissolved in an acetic acid solution (0.5 M) and mixed well. The mixture was centrifuged further at 10,000× *g* for 10 min to remove insolubilized pellets. The solubilized samples were transferred into a quartz cell. The spectral wavelengths were set, starting from 400 to 200 nm, and the solvent (0.5 M acetic acid) was used as a baseline.

#### 4.3.5. Attenuated Total Reflectance–Fourier Transform Infrared Spectroscopy (ATR–FTIR)

An FTIR spectrometer (Agilent Cary 630, Santa Clara, CA, USA) was applied to analyze the IR spectra of the extracted collagens (PSSC, PSBC and PSCC), and the method used was referred to in the previous work by Matmaroh et al. [31]. The lyophilized samples (around 5 mg) were placed onto the crystal cell of the spectrometer. All spectra were acquired with a resolution of 2 cm^−1^ throughout a wavenumber range of 4000–800 cm^−1^ for 32 scans against a background spectrum obtained from clean, empty cells at ambient temperature. The Agilent Microlab software tool was used to determine the spectra data.

#### 4.3.6. Determination of X-ray Diffraction (XRD)

XRD data of each pepsin-soluble collagen were collected using an X-ray diffraction apparatus (Rigaku Smart Lab^®^, Akishima, Japan), according to Chen et al. [48]. Lyophilized PSSC, PSBC and PSCC samples were scanned under an XRD machine with copper Kα as an X-ray source. The tube voltage and current were adjusted at 40 kV and 50 mA, respectively. The scanning range in all lyophilized samples was measured from 10° to 50° (2θ) with a speed of 0.06° per second.

#### 4.3.7. Determination of Differential Scanning Calorimetry (DSC)

The thermostability of PSSC, PSBC and PSCC was analyzed using a DSC apparatus (Perkin-Elmer, Model DSC7, Norwalk, CA, USA) based on the procedure by Kittiphattanabawon et al. [37]. Lyophilized collagens were rehydrated at a ratio of 1:40 (*w*/*v*) with de-ionized water. Rehydrates were then placed in a chiller for two days. Subsequently, the rehydrated fish collagens were weighed (between 5 mg and 10 mg) into an aluminum pan (Perkin-Elmer, Norwalk, CA, USA) and then tightly sealed. Before running the samples, the DSC machine was calibrated using indium as a standard. Furthermore, the sealed samples were scanned between 20 °C and 50 °C at a rate of 1 °C per minute. An empty pan was applied as a baseline. The maximum transition temperature (*T_max_*) was recorded from the thermogram’s endothermic peak, whilst the total denaturation enthalpy (Δ*H*) was obtained from the thermogram’s area.

#### 4.3.8. Field Emission Scanning Electron Microscopy (FESEM)

The morphological structure of freeze-dried collagens from lizardfish skin, bone and scales by aiding pepsin was evaluated under field emission scanning electron microscopy (JSM-7900F, JEOL, Tokyo, Japan). All collagen samples (PSSC, PBSC and PSCC) were sputter coated with gold for 5 min using a coater (JEOL JFC-1200, Tokyo Rikakikai Co., Ltd., Tokyo, Japan). Following coating, the samples were then imaged at 1000× magnification [26].

#### 4.3.9. Solubility Measurement

Solubility at pH 1.0 to pH 11.0 and various sodium chloride concentrations (0–60 g/L) were implemented, as in our previous work. For different pH solubilities, PSSC, PSBC and PSCC were dissolved with an acetic acid solution (0.5 M) and continuously stirred for 24 h in a cool room. Then, the solubilized samples were treated with various pH levels, from pH 1.0 to pH 11.0, and the prepared acidic (2.5 N HCl) and alkaline (2.5 N NaOH) solutions were used for pH adjustments. The pH-adjusted samples were stirred for around 2 h and centrifuged further at 8500× *g* for 20 min in a centrifuge (Eppendorf 5430R Refrigerated Centrifuge, Hamburg, Germany). For solubility in NaCl, about 5 mL of solubilized collagens were added into 5 mL of various NaCl concentrations and stirred for 30 min in a chiller using a magnetic stirrer (ST0707V2, Selangor, Malaysia). Subsequently, the treated samples were centrifuged at 8500× *g* for 20 min. Following centrifugation, the protein content was measured according to Lowry et al. [64], and bovine serum albumin (BSA) was used as a standard. The percentage of the relative solubility observed in both pH and NaCl treatments was determined using the following equation:

(3) $$\mathrm{Relative}\;\mathrm{solubility}\;\left(\%\right)=\frac{\mathrm{Current}\;\mathrm{concentration}\;\mathrm{of}\;\mathrm{protein}\;\mathrm{at}\;\mathrm{current}\;\mathrm{NaCl}\;\mathrm{or}\;\mathrm{pH}}{\mathrm{The}\;\mathrm{highest}\;\mathrm{concentration}\;\mathrm{of}\;\mathrm{protein}}\;\times \;100$$

### 4.4. Statistical Analysis

The experiments were conducted in triplicate using a completely randomized design. The collected data were presented as means with standard deviation, and the probability value of <0.05 was considered as significant. One-way ANOVA was applied and Duncan’s multiple range test was used to compare the means mean comparisons under SPSS Statistics version 28.0 (IBM Corp., Armonk, NY, USA).

## Figures and Tables

**Table 1:** Yield, Hyp, total collagen and color attributes of pepsin-soluble collagen.

Sample	Yield (%) Wet Weight	Hyp (mg/g)	Collagen (mg/g)	Color Parameters			
				L*	a*	b*	WI
PSSC	3.50 ± 0.11 ^a^	85.71 ± 0.13 ^a^	659.94 ± 0.99 ^a^	64.87 ± 3.19 ^b^	3.46 ± 0.38 ^a^	6.83 ± 0.73 ^b^	64.03 ± 3.24 ^b^
PSBC	3.26 ± 0.10 ^a^	84.85 ± 1.38 ^a^	653.33 ± 10.60 ^a^	79.91 ± 0.04 ^a^	0.31 ± 0.35 ^b^	3.59 ± 0.06 ^c^	79.58 ± 0.03 ^a^
PSCC	0.60 ± 0.06 ^b^	81.72 ± 0.47 ^b^	629.28 ± 3.61 ^b^	81.04 ± 0.45 ^a^	0.17 ± 0.13 ^b^	11.95 ± 1.34 ^a^	77.57 ± 0.97 ^a^

PSSC, PSBC and PSCC represent pepsin-soluble lizardfish skin, bone and scale collagens, respectively. Means with different lower-case letters in the same column are significantly different (*p* < 0.05).

**Table 2:** FTIR spectroscopy peak area and the assignment for the pepsin-soluble collagens.

Peak Type	Peak Description	Peak Location		
		PSSC	PSBC	PSCC
Amide A	N-H stretching coupled with hydrogen bond	3278.28 cm^−1^	3295.05 cm^−1^	3293.19 cm^−1^
Amide B	CH_2_ asymmetric stretching	2922.31 cm^−1^	2922.31 cm^−1^	2922.31 cm^−1^
Amide I	C=O stretching/hydrogen bond coupled with COO-	1636.34 cm^−1^	1636.34 cm^−1^	1636.34 cm^−1^
Amide II	N-H bend coupled with C-N stretching	1541.29 cm^−1^	1541.29 cm^−1^	1541.29 cm^−1^
Amide III	N-H bend coupled with C-H stretching	1233.78 cm^−1^	1233.78 cm^−1^	1237.51 cm^−1^

PSSC: pepsin-soluble skin collagen; PSBC: pepsin-soluble bone collagen; PSCC: pepsin-soluble scales collagen.

## Data Availability

The data presented in this study are available upon request from the corresponding author.
